# 

**Published:** 1893-05

**Authors:** 


### CONTENTS.

### COMMUNICATIONS.

Biographical Sketch of Dr. George Watt .... 209
Disinfectants .... 216
Removal of the Gasserian Ganglion .... 221

### SELECTIONS.

Dentition in Infants .... 223
Removal of a Foreign Body (Pin) from the Throat of a Child .... 227
Carbolic Acid in Surgery .... 228
Society Work .... 229
The Present Status of Corrosive Sublimate in Surgery .... 231
The Passage of Microbes Through the Skin .... 234
Dental Anæsthetics, etc .... 235
Reunion of Parts Severed from the Body .... 235
Bleaching Bones .... 237
Expectorant .... 238
Imitation of Gold .... 238
Overcrowded Professions .... 239
An Explosive Powder .... 239
Antiseptics .... 240
Solidified Chloroform .... 240
Gum Lancing .... 241
Amblyopia Due to Dental Irritation .... 242
Good Cause for Suicide .... 243
Carbolic Acid as an Antiseptic .... 243
Commencements .... 244

### NOTICES.

World's Columbian Dental Congress—Women Dentists .... 252
The World's Columbian Exposition .... 254
Columbian Dental Club, Chicago .... 255

### EDITORIAL.

The Specialist Before a General Medical Society .... 257
A New Dental College .... 259
A Change .... 259
American Medical Association .... 260
Death of Doctor W. W. Dawson, M. D. .... 260

